# Lightweight 3D CNN for MRI Analysis in Alzheimer’s Disease: Balancing Accuracy and Efficiency

**DOI:** 10.3390/jimaging11120426

**Published:** 2025-11-28

**Authors:** Kerang Cao, Zhongqing Lu, Chengkui Zhao, Jiaming Du, Lele Li, Hoekyung Jung, Minghui Geng

**Affiliations:** 1 College of Computer Science and Technology, Shenyang University of Chemical Technology, Shenyang 110000, China; caokerang@syuct.edu.cn (K.C.);; 2 Key Laboratory of Intelligent Technology of Chemical Process Industry in Liaoning Province, Shenyang 110142, China; 3 Computer Engineering Department, Paichai University, Daejeon 35345, Republic of Korea

**Keywords:** Alzheimer’s disease, lightweight 3D CNN, model pruning, multi-scale feature extraction, computational efficiency, image processing

## Abstract

Alzheimer’s disease (AD) is a progressive neurodegenerative disorder characterized by subtle structural changes in the brain, which can be observed through MRI scans. Although traditional diagnostic approaches rely on clinical and neuropsychological assessments, deep learning-based methods such as 3D convolutional neural networks (CNNs) have recently been introduced to improve diagnostic accuracy. However, their high computational complexity remains a challenge. To address this, we propose a lightweight magnetic resonance imaging (MRI) classification framework that integrates adaptive multi-scale feature extraction with structural pruning and parameter optimization. The pruned model achieving a compact architecture with approximately 490k parameters (0.49 million), 4.39 billion floating-point operations, and a model size of 1.9 MB, while maintaining high classification performance across three binary tasks. The proposed framework was evaluated on the Alzheimer’s Disease Neuroimaging Initiative dataset, a widely used benchmark for AD research. Notably, the model achieves a performance density(PD) of 189.87, where PD is a custom efficiency metric defined as the classification accuracy per million parameters (% pm), which is approximately 70× higher than the basemodel, reflecting its balance between accuracy and computational efficiency. Experimental results demonstrate that the proposed framework significantly reduces resource consumption without compromising diagnostic performance, providing a practical foundation for real-time and resource-constrained clinical applications in Alzheimer’s disease detection.

## 1. Introduction

Alzheimer’s disease (AD) primarily affects older adults and is a leading cause of cognitive impairment worldwide [1]. The disease is characterized by progressive deterioration of memory and executive functions, severely compromising patients’ daily living activities [2]. In its advanced stages, AD leads to extensive brain atrophy and functional decline, often resulting in loss of independence, behavioral disturbances, and heightened vulnerability to other health complications that contribute to mortality [3]. Therefore, early and accurate diagnosis of AD is of great clinical significance for timely intervention and disease management.

Clinically, AD progression is often categorized into three stages: normal cognition (NC), mild cognitive impairment (MCI), and AD dementia [4]. Accurate classification among these stages requires comprehensive diagnostic tools that integrate neuropsychological assessments, biomarker analyses, and neuroimaging techniques. Among these, magnetic resonance imaging (MRI) plays a particularly critical role by providing noninvasive, high-resolution visualization of structural brain alterations [5]. Deep learning-based analysis of MRI data has demonstrated considerable potential for automatic feature extraction and disease classification, offering improved consistency and scalability compared with manual or traditional machine learning methods.

In recent years, numerous Artificial Intelligence (AI)-based medical applications have achieved impressive predictive accuracy but often remain limited in scientific interpretability and clinical generalization. For instance, many deep learning models used for disease screening focus primarily on end-to-end classification accuracy without exploring the underlying neuropathological mechanisms or ensuring reproducibility across diverse populations [6,7]. Such approaches, while technically sound, contribute little to scientific understanding or real-world clinical translation. In contrast, the present study emphasizes interpretable and resource-efficient AI design by integrating adaptive multi-scale feature extraction and lightweight structural optimization, aiming to enhance both diagnostic relevance and scientific insight.

The application of deep learning, particularly three-dimensional 3D CNNs, has significantly advanced medical image analysis [8]. 3D CNNs perform volumetric convolutions that capture spatial context and structural dependencies within MRI data, enhancing the model’s ability to identify subtle pathological variations related to AD [9]. However, conventional 3D CNNs often require substantial computational resources and storage, limiting their deployment in real-world clinical settings where hardware constraints are common. These challenges highlight the need for models that not only achieve high diagnostic accuracy but also maintain computational efficiency.

Multi-scale convolutional techniques employ convolutional kernels of different sizes to capture fine-grained local features and broad contextual information simultaneously. Such methods enhance the model’s adaptability to varying anatomical structures and scales of pathological change [10]. In medical imaging, multi-scale feature extraction has been successfully applied to lesion detection, tissue segmentation, and disease classification. Nevertheless, integrating multi-scale information within a 3D CNN architecture often increases model complexity, leading to longer inference times and greater computational demands.

To address these challenges, this study presents a lightweight 3D CNN framework that combines adaptive multi-scale feature extraction with structural pruning and parameter optimization. The proposed architecture effectively reduces model redundancy, compresses network parameters, and lowers floating-point operations (FLOPs) while maintaining competitive classification performance. From the perspective of artificial intelligence, this work contributes to the advancement of efficient 3D deep learning paradigms by introducing a novel adaptive multi-scale mechanism that dynamically balances feature richness and computational cost. Unlike conventional architectures that prioritize accuracy at the expense of efficiency, our approach emphasizes the co-optimization of model compactness and diagnostic capability, aligning with current AI trends toward sustainable and deployable intelligence [11]. Furthermore, the lightweight design demonstrates how structural compression and kernel adaptivity can generalize beyond medical imaging, offering a transferable strategy for broader AI applications that require real-time processing under resource constraints.By significantly improving inference efficiency and reducing computational burden, the lightweight design makes advanced AD classification models more practical for clinical applications and real-time deployment. This work aims to bridge the gap between diagnostic accuracy and computational feasibility, promoting the broader adoption of AI-assisted early detection systems for Alzheimer’s disease.

Beyond clinical application, our framework contributes to AI paradigms by demonstrating how adaptive multi-scale feature extraction combined with lightweight structural optimization can achieve both high efficiency and interpretability. This approach exemplifies a shift toward resource-conscious, deployable, and scientifically meaningful AI architectures, supporting broader adoption in real-world settings.

## 2. Related Work

### 2.1. Deep Learning for Alzheimer’s Disease Classification

In recent years, deep learning has been widely applied to the automated classification of AD using neuroimaging data such as structural MRI and PET scans. Various 3D CNNs have been developed to capture spatial and contextual information from brain volumes, significantly improving diagnostic accuracy compared to traditional machine learning methods. For instance, multi-scale feature extraction and attention mechanisms have been incorporated to enhance feature representation and robustness in AD, MCI, and NC classification tasks [12]. Other studies have explored hybrid and ensemble frameworks that combine multiple 3D CNN models or integrate multimodal inputs to achieve improved performance [13,14]. Despite these advances, most existing networks are computationally intensive, with millions of parameters (i.e., the individual elements within weight matrices) and large memory footprints, which hinders their use in clinical or portable diagnostic environments [15].

### 2.2. Lightweight Networks in Medical Imaging

In general computer vision research, lightweight neural networks such as MobileNet, ShuffleNet, and EfficientNet have been introduced to reduce computational costs while maintaining competitive accuracy [16]. These architectures employ strategies such as depthwise separable convolution, channel pruning, and knowledge distillation to optimize efficiency [17]. In medical image analysis, lightweight models have gradually gained attention, particularly for applications that require real-time processing or operation on edge devices [18]. However, most of these studies have focused on 2D imaging tasks such as chest X-ray classification or ultrasound analysis [19].

### 2.3. Lightweight Models for Alzheimer’s Disease

In the context of AD, relatively few studies have explored lightweight 3D CNN architectures [20]. Existing works primarily emphasize accuracy improvement through complex network designs—such as multi-scale feature fusion, transformer-based modules, or cross-modal attention—rather than computational efficiency [21]. Consequently, the majority of AD classification models remain large and resource-intensive, limiting their applicability in practical healthcare environments. Furthermore, systematic evaluations of the trade-off between diagnostic accuracy and computational cost are scarce in this field [22]. Therefore, there is a clear need for lightweight, computation-efficient 3D CNN frameworks specifically tailored for AD diagnosis, enabling real-time and scalable clinical deployment.

Overall, the research directions that have demonstrated the greatest impact in AD classification include the development of multi-scale and attention-based 3D CNN architectures, the integration of lightweight and efficient network designs, and the exploration of transformer-based modules for improved interpretability and multimodal integration. Highlighting these paths not only reflects the current state-of-the-art but also emphasizes avenues likely to drive future advances in both AI methodology and clinical applicability.

To provide a rigorous basis for comparison, in this work we evaluate existing AD classification models not only in terms of predictive accuracy but also considering computational cost, model size, and inference efficiency. By systematically quantifying both performance and resource requirements, we use rigorous criteria to ensure fair and meaningful comparisons across different architectures and paradigms.

## 3. Models and Methods

MRI provides a non-invasive way to visualize brain structural features. The neuron density and spin density of brain tissues affect the MRI signal intensity, which is captured as a series of 2D slices forming a 3D volume. Each voxel corresponds to a specific spatial location, and its intensity reflects the local signal strength, typically normalized to ensure consistency across subjects. The preprocessed 3D MRI data serve as input to a CNN. The CNN extracts local spatial features through convolutional layers and enhances nonlinear representations via activation functions. The features are then flattened and fed into fully connected layers for classification, enabling the network to learn discriminative patterns from MRI scans for Alzheimer’s disease identification.

**Figure 1:**
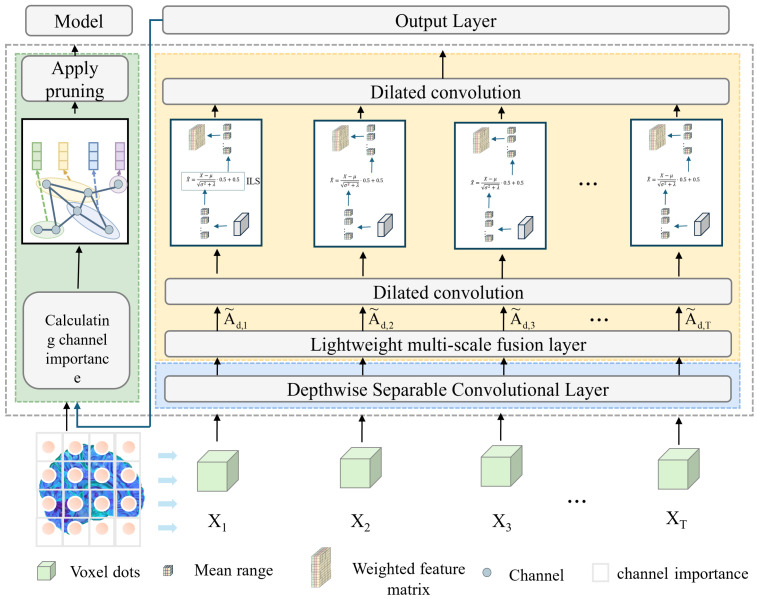
Schematic diagram of binary classification model training.

In medical image analysis, binary classification plays a fundamental role in distinguishing between different clinical conditions, such as separating patients with cognitive impairment from healthy controls. Compared to multi-class classification, binary classification tasks are often more practical in clinical settings, as they focus on specific diagnostic objectives and help clinicians make clearer decisions. However, deep learning models used for binary classification in medical imaging typically require large computational resources (For example, training a 3D CNN with millions of parameters may require GPUs such as NVIDIA RTX 4090 (24 GB VRAM) and several hours of computation per epoch.), which may limit their applicability in real-world scenarios where efficiency and deployment on limited hardware are essential. To address this challenge, lightweight models have gained increasing attention, as they reduce computational cost and memory usage while maintaining competitive accuracy, making them more suitable for clinical applications.The lightweight binary classification framework we designed is as follows:

The workflow of the proposed model begins with the loading and preprocessing of three-dimensional MRI data, where the input is a single-channel volumetric image. A 1 × 1 × 1 convolution layer is first employed for channel mapping and preliminary feature extraction, which reduces computational complexity while enhancing representation capacity. Subsequently, a Depthwise Separable Conv3D is introduced, in which spatial and channel convolutions are decoupled. This design significantly decreases the number of parameters and computational cost, while retaining the ability to capture local spatial patterns. To further strengthen feature representation, a lightweight multi-scale fusion module (LMF) block is integrated in the intermediate layers. Within this module, multi-scale convolutional branches with kernels of 3 × 3 × 3 and 5 × 5 × 5 operate in parallel to extract features under different receptive fields, followed by a Improved and lightweight channel attention mechanism that adaptively reweights and fuses these features, thereby enabling effective multi-scale information integration. The selection of 3 × 3 × 3 and 5 × 5 × 5 convolution kernels is based on experimental evidence that these kernel sizes provide the best trade-off between precision and efficiency. Smaller kernels (e.g., 1 × 1 × 1) tend to lose spatial detail, while larger ones (e.g., 7 × 7 × 7) greatly increase the computational burden. As demonstrated in Table 1 and Table 2, the combination of 3 × 3 × 3 and 5 × 5 × 5 achieves the optimal balance, maintaining high classification accuracy while ensuring the model remains lightweight. This design choice effectively supports real-time applicability without sacrificing representational capacity. At higher semantic stages, dilated convolutions are incorporated to expand the receptive field and capture richer contextual information without increasing the parameter count. In parallel, an improved lightweight Similarity-Aware Attention Module (SimAM), referred to as ILS, is adopted to model feature energy, thereby enhancing fine-grained discriminative features while suppressing redundancy. Finally, compact and discriminative feature representations are obtained through global average pooling and a fully connected layer, ensuring a balance between lightweight design and classification performance. As shown in Figure 1.

In deep learning frameworks utilizing MRI data, the intermediate representations are essential for precise classification outcomes [23]. To effectively model both detailed anatomical structures and broad contextual information in 3D brain volumes, we propose the LMF module. It combines multi-scale convolutional operations, hierarchical feature integration, and adaptive channel weighting to automatically emphasize the most relevant anatomical regions. Through the joint use of local and global cues, the LMF design enhances the model’s sensitivity to minor pathological variations—such as cortical thinning and hippocampal shrinkage—thereby improving diagnostic performance without increasing computational cost.

**Figure 2:**
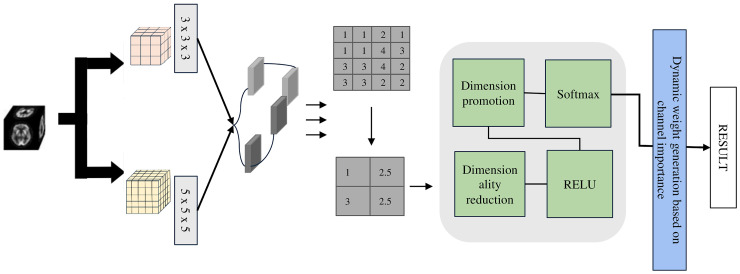
Overall architecture of the LMF module.

The LMF module (see Figure 2), developed based on extensive experiments (see Table 1) with multi-scale convolutions, employs parallel 3 × 3 × 3 and 5 × 5 × 5 depthwise separable convolution kernels to balance model lightweightness and classification accuracy. Depthwise separable convolutions separate spatial and channel-wise operations, providing a 1 × 1 × 1 receptive field while significantly reducing the number of parameters and computational cost, thus achieving a lightweight design. Specifically, the 1 × 1 × 1 convolution is used for channel information integration and efficient feature transformation, while the 3 × 3 × 3 and 5 × 5 × 5 convolutions capture medium- and large-scale structural features, respectively.

Specifically, the depthwise separable 3D convolution establishes the lightweight foundation of the model, and its formulation is expressed as follows:

(1) $$y_{c}\left(p\right)\;=\;\sum_{q\in \Omega }\mathrm{Conv}\left(w_{c}\left(q\right),x_{c}\left(p+q\right)\right)$$

(2) $$z_{k}\left(p\right)\;=\;\sum_{c=1}^{c_{in}}\mathrm{Conv}\left(v_{k,c},y_{c}\left(p\right)\right)$$

where *c* denotes the channel, Ω represents the receptive field of the convolution kernel, and $w_{c}$ corresponds to the convolution kernel of the c channel, $v_{k,c}$ represents the point-by-point convolution weight, which is used for cross-channel information fusion. Equation (1) represents performing a convolution on the input feature map $x_{c}$ at spatial position *p* over a local neighborhood defined by the offset *q*, producing an intermediate feature $y_{c}\left(p\right)$. Equation (2) linearly combines the intermediate features $y_{c}\left(p\right)$ across all channels using weights $v_{k,c}$, achieving channel-wise fusion and resulting in the final output feature $z_{k}\left(p\right)$. In short, the first represents spatial convolution, while the second represents channel fusion.

In the LMF block, we use different convolution kernels (For example, 1 × 1 × 1, 3 × 3 × 3, and 5 × 5 × 5 kernels extract multi-scale features across channels) to extract features in parallel. The formula can be written as:

(3) $$F_{i}\;=\;{\mathrm{Conv}}_{k_{i}}\left(X\right),i\;=\;1,\ldots ,S$$

where $k_{i}$ represents convolution kernels of different sizes, *S* is the number of scales, and the output $\left\{F_{1},F_{2},\ldots ,F_{S}\right\}$ represents multi-scale features. After obtaining the multi-scale features, they are concatenated and globally pooled, followed by feature reweighting through our designed lightweight adaptive weighting module, and its formulation is expressed as follows:

(4) $$z\;=\;\frac{1}{DHW}\sum_{d=1}^{D}\sum_{h=1}^{H}\sum_{w=1}^{W}\mathrm{Concat}\left(F_{1},F_{2},\ldots ,F_{S}\right)$$

Equation (4) represents concatenating multiple 3D feature maps $F_{1},F_{2},\ldots ,F_{S}$ along the channel dimension, and then computing the average over the entire spatial dimensions (Depth *D*, Height *H*, Width *W*), i.e., 3D global average pooling, to obtain a global feature for each channel. For example, for a feature map of size D=4,H=3,W=3, after concatenating two features, the number of channels at each position becomes 2. Averaging over all 4×3×3=36 positions yields a vector *z* of length 2, representing the globally pooled channel features.

(5) $$\alpha \;=\;\mathrm{Softmax}(W_{2}\delta \left(W_{1}z\right))$$

The Equation (5) indicates that the input vector *z* is first linearly transformed and passed through an activation function, and then Softmax converts the result into a probability distribution. The input can be any real-valued vector, and the output gives probabilities for each element summing to 1. For example, applying Softmax to the scores [2.0,1.0,0.1] results in [0.66,0.24,0.10], indicating the first class has the highest predicted probability.

(6) $$F\;=\;\sum_{i=1}^{S}\alpha_{i}\times F_{i}$$

where δ(·) is the ReLU(Rectified Linear Unit) activation function, and $\alpha =\{\alpha_{1},\ldots ,\alpha_{S}\}$ represents the fusion weights of multi-scale features.

The core idea of ILS is to model feature importance through an energy function and normalize it using Sigmoid. Its attention weight formula is:

(7) $$E_{ijk}\;=\;\frac{{\left(x_{ijk}-\mu \right)}^{2}}{\phi^{2}+\lambda },\;M_{ijk}\;=\;\sigma \left(E_{ijk}\right),\;Y\;=\;X⊙M$$

Compared to traditional SimAM, we omit the explicit energy function and instead adopt a normalization and translation scaling approach to stabilize the numerical distribution, and obtain weights through Sigmoid activation. This not only avoids additional steps in energy calculation but also ensures a more balanced feature distribution across different scales. its formulation is expressed as follows:

(8) $$\hat{X}\;=\;\frac{X-\mu }{\sqrt{\phi^{2}+\lambda }}\times 0.5+0.5,\;M\;=\;\sigma \left(\hat{X}\right),\;Y\;=\;X⊙M$$

$x_{ijk}$ represents the values of the input feature map at spatial positions (i,j,k); μ,σ are the mean and variance of the channel, respectively; λ is a smoothing term used to avoid zero division; ϕ(·) is the Sigmoid function; *M* is the attention mask, *Y* is the weighted output, $\hat{X}$ is the normalized feature map, and the range is shifted and scaled to around [0,1].

The expansion convolution of the last two layers is used to expand the receptive field, and its formula is:

(9) $$y\left(d,h,w\right)\;=\;\sum_{(i,j,l)\in \Omega }x\left(d+r\times i,h+r\times j,w+r\times l\right)\times w\left(i,j,l\right)$$

Equation (9) defines the 3D dilated convolution operation. Specifically, x(d,h,w) denotes the input 3D feature map, where *d*, *h*, and *w* represent the depth, height, and width indices, respectively. The convolution kernel weights are denoted by w(i,j,l) at offset (i,j,l)∈Ω, and *r* indicates the dilation rate that controls the spacing between kernel elements. For example, when using a 3×3×3 kernel with a dilation rate of r=1, each output voxel y(d,h,w) is obtained by performing element-wise multiplication between the corresponding 3×3×3 region of the input *x* and the kernel weights *w*, followed by summation. This operation enables the model to capture local spatial dependencies in 3D volumetric data.

In order to make the model more lightweight, we introduce the asymptotic pruning method, which sets the redundant channels of the model to zero during training. The threshold projection formula for pruning is as follows:

(10) $$W^{\prime }=W⊙1\left(|\gamma |\geq \tau (r)\right),\tau \left(r\right)={\mathrm{Quantile}}_{r}\left(|\gamma |\right)$$

Among them, *W* is the weight arranged by the convolutional layer according to the output channel (corresponding one-to-one to the corresponding BatchNorm (BN) channel), γ corresponds to the channel scaling parameter vector. The uantile r(|γ|) of BN is used such that channels with a proportion *r* satisfying |γ|<τ are pruned. 1(·) is the indicator function: it takes 1 if the condition is met, otherwise 0. ⊙ represents element-wise multiplication broadcasted by channel.

## 4. Materials and Experiments

The experiments were conducted in a Python 3.9 environment with PyTorch 2.1 and CUDA 11.8 on a high-performance computing platform equipped with an NVIDIA RTX 4090 GPU (24 GB; NVIDIA Corporation, Santa Clara, CA, USA) and an Intel Core i9-14900 processor (Intel Corporation, Santa Clara, CA, USA).

The model employs a 3D convolutional network architecture and was trained for a total of 100 epochs. Each batch contains 4 samples, and gradient accumulation is applied to achieve an effective batch size of 16, balancing memory usage and training stability. The loss function is Label Smoothing Cross-Entropy LabelSmoothingLoss, smoothing = 0.1), and a sparsity regularization on BatchNorm γ ($\lambda \;=\;1\times 10^{-4}$) is applied to constrain model complexity and enhance lightweight efficiency. The optimizer is SGD (Stochastic Gradient Descent) with a learning rate of 0.005, momentum of 0.9, and weight decay of $1\times 10^{-4}$, while a ReduceLROnPlateau scheduler adjusts the learning rate according to the training loss. Progressive pruning is performed every 5 epochs (prune ratio per step =0.05) to remove redundant channels and further compress the model. During training, both training and validation losses and accuracies are recorded, and a checkpoint of the model is saved at the end of each epoch to ensure reproducibility while maintaining model performance.

The dataset we are using is from Alzheimer’s Disease Neuroimaging Initiative (ADNI), The ADNI is one of the most representative and influential publicly available databases for Alzheimer’s disease research. Launched in the United States in 2004, its goal is to promote early diagnosis and intervention of AD through multi-center, multi-modal longitudinal studies. The dataset includes a wide range of multimodal data from diverse participants, such as structural MRI, functional MRI, PET imaging, genetic information, biomarkers, and clinical assessment scales, covering individuals across different stages including NC, MCI, and AD. ADNI not only provides standardized, high-quality data resources for clinical research but also serves as an important platform for developing and validating machine learning and deep learning methods in early diagnosis and progression prediction of AD.

The ADNI database offers preprocessed MRI data to ensure consistency and reliability in subsequent analyses. In this work, several key preprocessing steps were applied to the acquired images. First, co-registration was used to align scans from different sessions or imaging modalities into a unified coordinate space, minimizing positional variations. Frame averaging was then performed to suppress random noise and enhance the signal-to-noise ratio by merging multiple acquisitions into a single image. Standardization converted all images to a uniform resolution and format, enabling cross-subject comparison. AC–PC correction adjusted brain orientation along the anterior commissure–posterior commissure axis, ensuring anatomical alignment across participants. Intensity normalization further rescaled voxel values to reduce scanner- or protocol-related variability. Skull stripping removed non-brain tissues such as scalp and skull, improving the accuracy of brain structure analysis. Finally, Gaussian smoothing was applied to suppress high-frequency noise and emphasize subtle structural differences by further enhancing signal quality.

**Figure 3:**
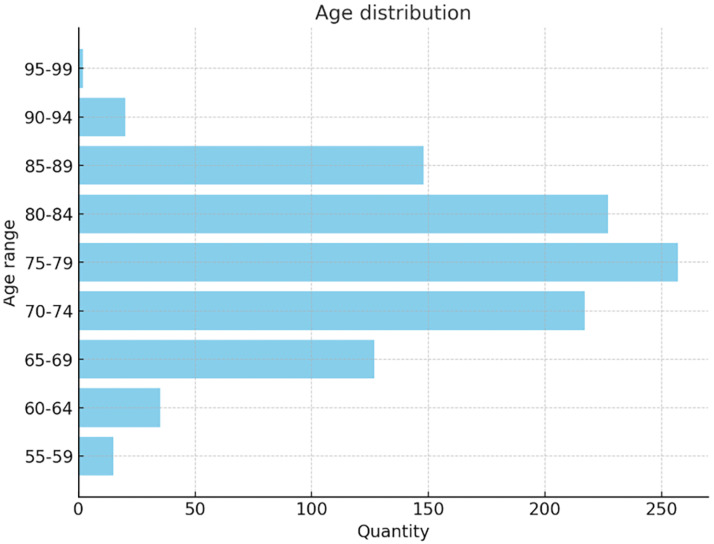
Dataset information diagram.

**Figure 4:**
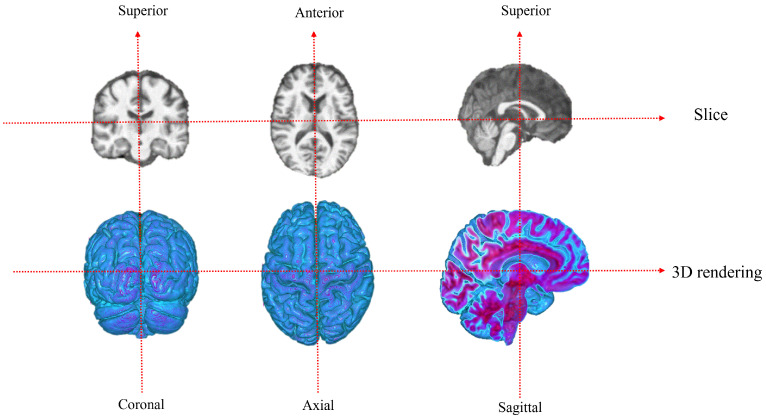
Dataset MRI visualization.

The dataset used in this study includes 1012 participants (555 males and 457 females, aged 55–90), covering a broad spectrum of cognitive conditions. A total of 2174 MRI images were classified into AD (443), MCI (365), and NC (1366), The age distribution in the dataset is shown in Figure 3:

The 3D rendering three views of the dataset are shown in Figure 4:

To address the issue of data imbalance, several complementary strategies were employed to enhance model generalization and minimize bias toward dominant categories. These included a five-fold cross-validation scheme, synthetic sample generation, and balanced resampling.

During the five-fold validation process, the dataset was partitioned into five equal subsets, and the training procedure was repeated five times. In each iteration, one subset served as the testing set, while the remaining subsets were used for model training and validation.

To ensure balanced learning, oversampling of minority classes was performed to equalize class distributions, and data augmentation was applied to further expand the training set. Given the volumetric characteristics of 3D MRI data, strong spatial transformations—such as large rotations or translations—were avoided, as they could distort structural integrity and destabilize the network. Instead, mild yet effective augmentations, including Gaussian noise injection and gamma correction, were adopted.

By integrating cross-validation, balanced resampling, and augmentation, the proposed approach effectively mitigated class imbalance and improved the reliability, robustness, and stability of the model evaluation process.

To evaluate the model’s lightweight characteristics, we selected four indicators from three core dimensions: structure, computation, and storage. These include the Number of Parameters, FLOPs (Floating Point Operations), Model Size, and Performance Density. The number of parameters reflects the structural complexity of the model, with fewer parameters indicating a more compact and efficient architecture for training and deployment. FLOPs measure the computational cost of a single forward pass, where lower values correspond to faster inference and better suitability for real-time or resource-constrained scenarios. Model size directly represents storage and memory requirements, with smaller sizes facilitating deployment on embedded or mobile devices. In addition, Performance Density—defined as the ratio of classification accuracy to the number of parameters—serves as an integrated metric that balances predictive performance and computational efficiency. Together, these four metrics provide a comprehensive evaluation of the model’s lightweight characteristics and its practical applicability in clinical settings.

1. Number of computational parameters (Para)

Measuring the total number of trainable parameters in a model is a fundamental indicator of lightweighting. The formula is as follows:

(11) $$\mathrm{Para}\;=\;\sum_{l=1}^{L}\left(k_{l}\times k_{l}^{\prime }\times c_{\mathrm{in}}^{\left(l\right)}\times c_{\mathrm{out}}^{\left(l\right)}+c_{\mathrm{out}}^{\left(l\right)}\right)$$

where *L* is the total number of convolutional or fully connected layers, $k_{l}$ is the kernel size of the *l*-th layer, $c_{in}^{\left(l\right)},c_{out}^{\left(l\right)}$ are the number of input and output channels of the *l*-th layer.

2. Floating Point Operations (FLOPs)

Measure the number of floating-point operations required for a model to perform forward inference once, reflecting the computational complexity. The FLOPs calculation formula for convolutional layers:

(12) $$\mathrm{FLOPs}\;=\;2\times H_{\mathrm{out}}\times W_{\mathrm{out}}\times D_{\mathrm{out}}\times C_{\mathrm{in}}\times K_{H}\times K_{W}\times K_{D}\times C_{\mathrm{out}}$$

Among them, $H_{out}$, $W_{out}$, $D_{out}$ are the output feature map sizes; $C_{in}$, $C_{out}$ are the number of input/output channels; $K_{H}$, $K_{W}$, $K_{D}$ are the size of the convolution kernel.

3. Model Size (MS)

Measuring the size of storage space or memory occupied by model files directly affects deployment efficiency. The formula is as follows:

(13) ModelSize(Bytes)=Para×b

*b* is the number of bytes occupied by each parameter (such as 4 bytes for 32-bit floating-point numbers).

4. Performance Density (PD)

To quantitatively evaluate the trade-off between model performance and complexity, we introduce a metric termed PD, defined as:

(14) $$\mathrm{PD}\;=\;\frac{\mathrm{ACC}}{\mathrm{Para}/10^{6}}\times 100$$

where ACC denotes the classification accuracy (in percentage), and Para represents the number of parameters in the model. The PD reflects how much accuracy is achieved per million parameters, serving as a measure of parameter efficiency. Although not a standard metric, it provides an intuitive quantification of the performance–complexity balance. The unit of PD can be interpreted as “% per million parameters (% pm)”, analogous to the “parts per million (ppm)” concept used in spectroscopy. Similar ideas of efficiency metrics have been discussed in prior works such as EfficientNet [24] and MobileNetV3 [25].

The classification ACC is defined as follows:

(15) $$\mathrm{ACC}\;=\;\frac{T_{p}+T_{n}}{T_{p}+T_{n}+F_{p}+F_{n}}$$

where $T_{p}$, $T_{n}$, $F_{p}$, and $F_{n}$ denote the numbers of true positives, true negatives, false positives, and false negatives, respectively. This metric measures the overall proportion of correctly classified samples in the dataset.

We also selected two other important accuracy indicators in medicine: SEN and SPE to reflect the degree of change with model lightweighting, and F1 score to reflect the handling of dataset imbalance.

1. Sensitivity (SEN)

Measures the ability of the model to correctly identify positive samples, i.e., the proportion of actual positive samples that are correctly predicted:

(16) $$\mathrm{SEN}\;=\;\frac{TP}{TP+FN}$$

2. Specificity (SPE)

Measures the ability of the model to correctly identify negative samples, i.e., the proportion of actual negative samples that are correctly predicted:

(17) $$\mathrm{SPE}\;=\;\frac{TN}{TN+FP}$$

3. F1 Score

F1 Score is defined as the harmonic mean of two core classification metrics—Precision and Recall. Its primary purpose is to comprehensively evaluate a model’s performance on the positive class, with particular value in scenarios involving imbalanced datasets (e.g., fraud detection, rare disease diagnosis, where positive samples are far fewer than negative ones). Where F1 sore is defined as:

(18) $$F1\;\mathrm{Score}\;=\;2\times \frac{\mathrm{Precision}\times \mathrm{Recall}}{\mathrm{Precision}+\mathrm{Recall}}$$

Before performing multi-scale convolution, we need to determine the type and number of convolution kernels, which requires balancing the accuracy and lightweight of multi-scale convolution. Considering the practical goal, we chose the ADvsMCI model, which is more relevant to early classification of Alzheimer’s disease, for the experiment. The experiment is shown in Table 1 and Table 2.

It can be observed that the choice of convolution kernels has no significant impact on classification accuracy. In particular, the 1 × 1 × 1 kernel contributes little additional benefit, which may be attributed to the fact that depthwise convolutions inherently provide a receptive field similar to that of a 1 × 1 × 1 kernel, thereby diminishing its effect. In contrast, larger kernels (e.g., 7 × 7 × 7) are capable of capturing broader contextual information but incur a much higher cost in terms of model lightweighting, substantially increasing both parameter count and computational overhead. Taking accuracy and efficiency into account, we removed the 7 × 7 × 7 kernel in our design and replaced it with lightweight dilated convolutions, which expand the receptive field without significantly increasing computational burden. This allows the model to maintain compactness while preserving the representational benefits of larger kernels. As shown in Table 1 and Table 2, dilated convolutions achieve the best accuracy while simultaneously meeting lightweighting objectives, thereby validating the effectiveness and practicality of this strategy.

The experiments were conducted using a lightweight 3D CNN model. This model is built around depthwise separable convolutions, with an initial channel size of 24, and integrates multi-scale convolutional kernels (3×3×3 and 5×5×5) together with LMF. A parameter-free ILS attention mechanism was further incorporated to enhance feature representation. To achieve model lightweighting, a sparsity regularization term based on BN γ weights ($\lambda \;=\;1\times 10^{-4}$) was added during training, along with a progressive pruning strategy: approximately 5% of less important channels were pruned every 5 epochs.

For the training configuration, the optimizer was SGD with a learning rate of 0.01, momentum of 0.9, and weight decay of $1\times 10^{-4}$. The classification loss function was CrossEntropyLoss (reduction = “mean”), with label smoothing (ε=0.1) applied in some experiments to improve generalization. The learning rate scheduling strategy employed ReduceLROnPlateau, which decayed the learning rate by a factor of 0.1 if the validation loss did not decrease for 10 consecutive epochs, with verbose mode enabled for monitoring.

A five-fold cross-validation was performed, and the results of the ablation experiments targeting model lightweighting are reported in Table 3. The performance density PD, calculated as the average value across the three binary classification tasks (AD vs. MCI, MCI vs. NC, and AD vs. NC), is also included to comprehensively reflect the overall balance between model accuracy and computational efficiency.

As shown in the table, the proposed lightweight strategies significantly reduce the model complexity at different levels. Compared with the original 3DCNN, pruning reduces the number of parameters from 35.39 M to 19.21 M, decreases FLOPs from 311 G to 169 G, and compresses the model size by nearly 46%, indicating that pruning can effectively shrink the network and eliminate redundant computations. By introducing depthwise separable convolution, the parameter count is further reduced to 0.92 M, while FLOPs and model size drop to 8.07 G and 3.51 M, respectively, accounting for only about 2.6% of the basemodelL (remove pruning and depthwise separable convolution), which demonstrates that depthwise convolution makes the most significant contribution to reducing computational and storage overhead. When depthwise separable convolution is combined with pruning, the model reaches its lowest complexity, with only 0.49 M parameters, 4.39 G FLOPs, and a model size of 1.91 M, corresponding to approximately 98.6% reduction compared with the basemodelL. Overall, depthwise convolution serves as the primary driver of model lightweighting, while pruning further removes redundant channels on this basis. The two methods are highly complementary, enabling the model to achieve substantial reductions in computation and storage costs while maintaining stable classification performance. Since the inference latency is closely related to the number of floating-point operations, the drastic reduction in FLOPs from 311 G to 4.39 G directly translates into a significant acceleration in inference. In practical deployment, this reduction corresponds to more than a 70× theoretical speed-up and achieves an average inference time of less than 0.1 s per MRI scan. Such efficiency highlights the model’s excellent real-time applicability, indicating its potential for rapid and scalable clinical screening, even on edge devices or resource-limited systems. Thus, the proposed approach provides a practical solution for brain MRI diagnosis in resource-constrained scenarios.

**Figure 5:**
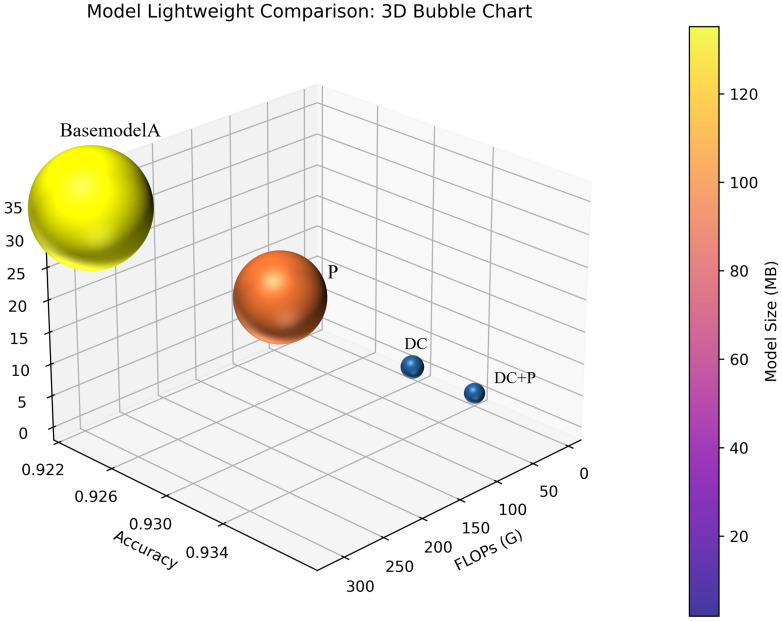
Model lightweighting and accuracy change diagram.

Moreover, the ratio between model performance and its parameter size, increases sharply with the introduction of lightweight strategies. Specifically, PD rises from 2.61 in the basemodelL model to 189.87 in the combined approach, reflecting that the proposed methods not only reduce computation and storage requirements but also greatly improve the efficiency of parameter utilization.In addition, the accuracy change of the overall lightweight model is shown in Figure 5.

It can be observed that the basemodelL is located in the upper-left region, with the highest number of parameters and FLOPs, as well as the largest bubble size, indicating the greatest storage and computational cost. The Pruning (P) model significantly reduces the number of parameters and model size while maintaining nearly unchanged accuracy, though the reduction in FLOPs is relatively limited. The Depthwise Convolution (DC) model and the combined DC+P model demonstrate substantial advantages in both FLOPs and parameter reduction, with much smaller bubble sizes that reflect minimal storage requirements; in particular, the DC+P model achieves the optimal lightweight performance. Notably, although different lightweight strategies lead to substantial differences in model complexity, the accuracy remains almost unchanged, suggesting that the proposed approach can achieve considerable compression and acceleration without compromising classification performance.

**Figure 6:**
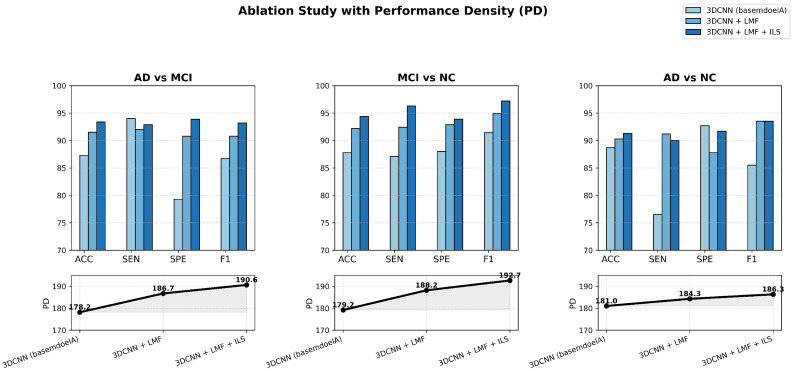
Model module ablation experiment and changes in performance density.

To further validate the effectiveness and efficiency of the proposed lightweight 3D CNN, ablation experiments were conducted across three binary classification tasks—AD vs. MCI, MCI vs. NC, and AD vs. NC—using MRI data. The results aim to examine the individual and combined contributions of the LMF and ILS modules to model performance and computational efficiency. Figure 6 presents a comprehensive visualization of these ablation outcomes, where bar charts and PD curves jointly illustrate how each enhancement progressively improves classification accuracy and efficiency while preserving the model’s lightweight nature.

The figure clearly illustrates the ablation results of the proposed lightweight MRI-based Alzheimer’s disease classification model across three comparative tasks: AD vs. MCI, MCI vs. NC, and AD vs. NC. Through bar charts and PD curves, it intuitively demonstrates the progressive improvements achieved as the 3DCNN model successively integrates the LMF and ILS modules, yielding consistent gains in ACC, SEN, SPE, and F1 score.

It is worth noting that our model performs best in MCI vs. NC classification and poorly in AD vs. NC classification. This result can be reasonably explained by the inherent heterogeneity of AD pathology and the subtle but consistent structural alterations observed in MCI. In AD patients, the atrophy pattern varies significantly across individuals, making the discriminative boundary more diffuse. In contrast, MCI subjects exhibit more homogeneous early-stage abnormalities, which can be effectively captured by the proposed LMF and ILS that focus on fine-grained spatial variations. Moreover, the relatively lower PD in AD vs. NC further reflects the higher intra-class variability and complexity of AD data, highlighting the robustness of our model when facing heterogeneous disease patterns.

Across all tasks, the PD values exhibit a stable upward trend from the basemodelA (remove LMF and ILS modules) to the enhanced configurations, confirming that each component contributes to both higher predictive accuracy and improved efficiency. This indicates that the proposed model can maintain strong performance while substantially reducing complexity. For instance, the enhanced model achieves comparable or even better accuracy with fewer parameters and shorter inference time—requiring less GPU memory and computation per scan—thereby clearly demonstrating lower computational cost. Overall, the model achieves an excellent balance between efficiency and precision, validating the effectiveness of the proposed lightweight design.

The ablation results further reveal that the LMF and ILS modules play complementary roles in performance enhancement. LMF captures multi-scale structural information, improving the network’s capacity to distinguish subtle brain pattern variations, while ILS strengthens attention to key lesion regions, enhancing discriminative focus. The improvements are particularly pronounced in the MCI vs. NC classification, indicating the model’s strong potential for early detection of mild cognitive impairment. Overall, the proposed lightweight 3D CNN achieves stable and interpretable gains across all tasks, proving its effectiveness, compactness, and practicality for efficient MRI-based Alzheimer’s disease diagnosis.

Furthermore, the experimental results for SEN and SPE indicate that the issue of dataset imbalance has been effectively mitigated. In classification tasks, if one class has significantly more samples than others, the model tends to be biased toward the majority class, leading to notable discrepancies between SEN and SPE and a decline in overall model performance, particularly for minority classes. By employing strategies such as data augmentation, oversampling, and cross-validation, this study successfully balanced the sample distribution across classes. As a result, the model demonstrates stable recognition performance for all categories, maintaining a high F1 score and further confirming its robustness in handling imbalanced datasets.

To assess whether the proposed model’s improvements over the baseline were statistically significant, a one-sample *t*-test was performed. This test evaluates whether the mean accuracy of the proposed model across five cross-validation folds is significantly higher than the baseline average, taking into account the variability of the folds. A *p*-value less than 0.05 indicates that the observed improvement is unlikely to occur by chance, the formula for the *t*-test is as follows:

(19) $$t\;=\;\frac{\bar{x}-\mu_{0}}{s/\sqrt{n}}$$

where $\bar{x}$ is the mean accuracy of the proposed model across *n* cross-validation folds, $\mu_{0}$ is the baseline model’s average accuracy, *s* is the standard deviation of the proposed model’s accuracies across folds, and *n* is the number of folds. A *p*-value less than 0.05 indicates that the improvement over the baseline is statistically significant, the final results are shown in Table 4.

The proposed model achieved higher mean accuracies than the baseline across all tasks. Single-sample *t*-tests show that these improvements are statistically significant (*p* < 0.01), confirming the model’s consistent robustness.

To further verify the robustness of our model, we also tested it on an external subset of the Open Access Series of Imaging Studies (OASIS) dataset (Includes 204 AD, 243 MCI, and 371 NC.). We used the same preprocessing on the MRI data from OASIS and then directly tested and fine-tuned the model (For each classification, 30 OASIS MIR images are used as input. The preceding convolutional layers are frozen, the final classification head is fine-tuned, and then testing is performed.). The results are shown in Table 5.

As shown in Table 5, the model trained on ADNI achieved accuracies of 86.0%, 86.4%, and 87.3% for AD vs. NC, AD vs. MCI, and MCI vs. NC, respectively, when directly tested on OASIS. Compared to the original ADNI test performance (91.3%, 93.4%, and 94.4%), this corresponds to a decrease of approximately 5.3, 7.0, and 7.1 percentage points, which is consistent with typical cross-dataset performance drops reported in the literature [26]. The decline can be attributed to domain shifts arising from differences in scanning protocols, preprocessing pipelines, and sample distributions.

After fine-tuning the model with a small number of OASIS samples, the accuracies improved to 88.7%, 89.1%, and 89.7%, yielding gains of 2.7, 2.7, and 2.4 percentage points over direct testing. The variations in performance drop among tasks reflect the intrinsic difficulty and feature separability: AD vs. NC has more distinct anatomical differences, resulting in a smaller decline, whereas MCI-involved tasks have subtler distinctions and slightly larger drops. The consistent improvement after fine-tuning demonstrates that the adaptation strategy effectively mitigates domain shifts across tasks.These results indicate that our model possesses strong cross-dataset generalization: even when applied to a different MRI dataset, the model can achieve high classification performance with minimal fine-tuning, validating the transferability and robustness of the learned features.

**Figure 7:**
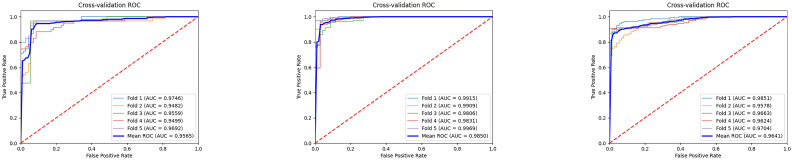
Five-fold cross-validation ROC curve of the model.

**Figure 8:**
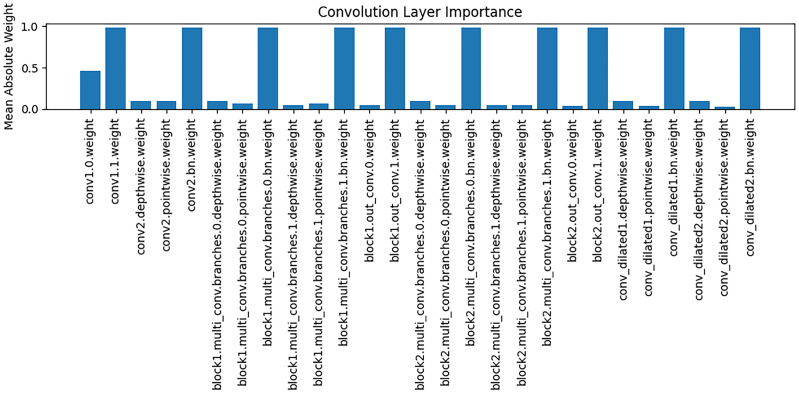
Model convolutional layer importance graph.

**Figure 9:**
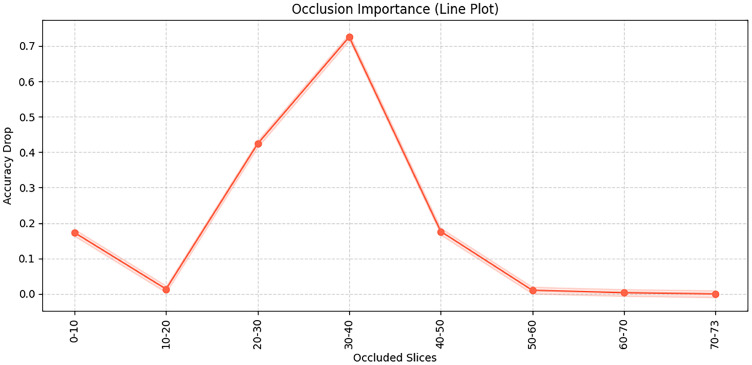
Model channel shielding test diagram.

The Figure 7 illustrates the ROC curves and average results of three binary classification tasks under five-fold cross-validation. It can be observed that the ROC curves of all three tasks are tightly clustered in the upper-left corner, indicating stable and superior classification performance. The average AUC values all exceed 0.95, with the MCI vs. NC task achieving the most prominent result, reaching an average AUC of 0.9850, which demonstrates the high reliability of the model in distinguishing mild cognitive impairment from normal controls. The average AUC values for the AD vs. MCI and AD vs. NC tasks are 0.9565 and 0.9641, respectively, also reflecting strong discriminative capability. Overall, these results indicate that the proposed model exhibits good generalization ability and stability across different classification tasks, providing strong support for the auxiliary diagnosis of Alzheimer’s disease at different stages. Furthermore, we conducted extended evaluations of the model’s interpretability, as shown in Figure 8 and Figure 9.

The Figure 8 presents an analysis of the importance of weights across different convolutional layers, with the vertical axis representing the mean absolute value of the weights. It can be observed that different convolutional layers play distinct roles in feature extraction: some layers (e.g., conv1.0.weight) exhibit relatively high weight magnitudes, indicating that these layers contribute more significantly to the extraction of key features during modeling; whereas other layers have weight magnitudes close to zero, suggesting a limited role in feature representation. These findings support the interpretability of the model, implying that the reliance on feature extraction varies across stages, and that critical convolutional layers play a central role in capturing Alzheimer’s disease–related brain structural features.

This Figure 9 illustrates the results of an occlusion test, showing the importance of different brain slices for the model’s classification decisions. Specifically, the occlusion test is conducted by systematically masking consecutive slices of the input 3D MRI volume and observing the resulting changes in classification accuracy, thereby revealing which slices are most critical for the model’s predictions. It can be observed that occluding slices 30–40 leads to the most significant accuracy drop, indicating that this region contains critical discriminative information. Similarly, occlusion of slices 20–30 also has a noticeable impact. In this study, each MRI scan consists of 73 axial slices (from inferior to superior), and this number is kept constant for all subjects after preprocessing. The “slices” refer to 2D cross-sectional images of the brain along the axial plane, corresponding roughly to different anatomical regions from the cerebellum and brainstem (lower slices) to cortical regions (upper slices). In contrast, occluding slices in the ranges of 0–20 and after 50 has relatively little effect on model performance, suggesting that these regions contribute less to the decision-making process. Therefore, the occluded slices between 20–40 mainly correspond to middle cerebral regions such as the hippocampus and temporal lobes, which are known to be closely associated with Alzheimer’s disease pathology.

## 5. Discussion

To demonstrate the effectiveness of the proposed lightweight 3D CNN model, we compared it with several representative compact 3D networks, including ResNet3D-Small [27], MobileNet3D [28], ShuffleNet3D [29] and Swin-UNETR [30]. Table 6 summarizes the classification performance, model complexity, and PD of each model. While all models achieve competitive accuracy, our lightweight network exhibits the highest PD, indicating superior utilization of parameters. Moreover, it maintains a minimal number of parameters and FLOPs, highlighting its efficiency and suitability for deployment in resource-constrained clinical environments.

Ours model achieves a strong balance between accuracy and efficiency in AD classification. With an accuracy of 93.1% and an F1 score of 0.93, it performs comparably to the much larger Swin-UNETR model, while maintaining a significantly smaller parameter count (0.49 M) and lower computational cost (4.39G FLOPs), resulting in the highest performance density (PD = 189.87) among the compared lightweight models. This demonstrates its effectiveness in utilizing computational resources efficiently. However, its accuracy is slightly lower than that of Swin-UNETR, indicating that very large models may still offer marginal gains, and further improvements could be explored to enhance predictive performance without compromising efficiency.

Comparative experiments are essential for objectively verifying the effectiveness and superiority of a proposed method. By evaluating performance against existing approaches or different model configurations, such experiments demonstrate improvements in accuracy, robustness, and efficiency, thereby strengthening the reliability of the research findings. In this study, the accuracy comparison experiments serve as a supplementary validation to the proposed lightweight framework, further confirming its effectiveness beyond structural simplification. The detailed classification accuracy results are presented in Table 7, Table 8 and Table 9, while the corresponding lightweight performance comparisons are summarized in Table 10.

These four tables comprehensively demonstrate the advantages of the proposed method in both classification performance and lightweight design. Specifically, Table 7, Table 8 and Table 9 correspond to the three common binary classification tasks: AD vs. NC, AD vs. MCI, and MCI vs. NC. It can be seen that the proposed 3DCNN model achieves the best or near-best results across these tasks. For example, in the AD vs. NC task, the proposed method outperforms traditional SVM and DenseNet models in both accuracy and F1 score; in the AD vs. MCI task, compared with CNN and DenseNet, the method shows clear advantages in accuracy and specificity; in the more challenging MCI vs. NC task, the method not only achieves significant improvements in accuracy and sensitivity but also reaches an F1 score of 0.972, indicating more precise and robust identification of key lesion regions.

Finally, Table 11 shows more detailed information about the comparison groups.

## 6. Conclusions

Overall, this paper describes a lightweight, multi-scale 3D CNN framework that not only addresses the early prediction of Alzheimer’s disease but also introduces methodological innovations relevant to AI research. The framework is evaluated on multiple binary classification tasks, demonstrating both effectiveness and efficiency.

The model is designed to be inherently lightweight, extensively using depthwise separable convolutions to significantly reduce parameters and computational cost. In addition, it incorporates BN γ sparsity regularization and channel-wise pruning, providing a systematic strategy for removing redundant channels and optimizing network efficiency. These techniques exemplify practical innovations in resource-efficient deep learning, which are broadly applicable beyond the current medical imaging context.

To effectively extract features from brain MRI scans, the model employs novel multi-scale attention convolution modules (LMF + ILS). Multi-scale convolutions capture structural information at different spatial resolutions, while the adaptive fusion module dynamically generates weights to emphasize the most informative scales. The ILS attention mechanism further enhances salient regions, improving the model’s sensitivity to subtle structural variations. Together, these modules represent a contribution to attention-based multi-scale feature learning in AI.

Nonetheless, some limitations remain. While the compact design ensures high efficiency, it may slightly limit the capture of very subtle features. Additionally, the model’s reliance on labeled MRI data suggests future extensions could explore multi-scale learning with multi-modal or unsupervised data, potentially further advancing AI paradigms. The model’s performance relies heavily on labeled MRI data for training, and future work could extend it to multi-scale learning using multi-modal, unsupervised data.

## Figures and Tables

**Table 1:** The Influence of Convolutional Kernel Selection on Accuracy (%).

Convolutional Kernel Combination	ACC	SEN	SPE
1×1×1, 3×3×3, 5×5×5	91.2	91.5	90.0
3×3×3, 5×5×5	91.5	91.5	91.4
3×3×3, 5×5×5, 7×7×7	92.5	92.4	92.8
5×5×5, 7×7×7	92.2	92.4	91.4
3×3×3, 5×5×5, Dilated convolution	93.2	93.3	92.9

**Table 2:** The influence of convolutional kernel selection on model lightweighting.

Convolutional Kernel Combination	Para	FLOPs	MZ
1×1×1, 3×3×3, 5×5×5	0.52 M	4.89 G	2.03 M
3×3×3, 5×5×5	0.49 M	4.39 G	1.91 M
3×3×3, 5×5×5, 7×7×7	0.57 M	6.30 G	2.22 M
5×5×5, 7×7×7	0.54 M	5.68 G	2.08 M
3×3×3, 5×5×5, Dilated convolution	0.49 M	4.38 G	1.91 M

**Table 3:** Experimental table for lightweight model ablation.

Model Design	Para	FLOPs	MZ	PD
3DCNN (basemodelL)	35.39 M	311 G	135.21 M	2.61
3DCNN + Pruning	19.21 M	169 G	73.29 M	4.81
3DCNN + Deconvolution	0.92 M	8.07 G	3.51 M	100.98
3DCNN + Deconvolution + Pruning	0.49 M	4.39 G	1.91 M	189.87

**Table 4:** Comparison of mean accuracies between proposed model and basemodel across classification tasks.

Task	Proposed Mean	Baseline Mean	t-Value	*p*-Value
AD vs. MCI	0.9333	0.873	7.98	0.0008
AD vs. NC	0.9126	0.887	4.15	0.007
MCI vs. NC	0.9444	0.878	6.30	0.0015

**Table 5:** Classification accuracies under different training strategies.

Strategy	AD vs. NC (%)	AD vs. MCI (%)	MCI vs. NC (%)
Original Testing	91.3	93.4	94.4
Direct Classification	86.0	86.4	87.3
Fine-tuning	88.7	89.1	89.7

**Table 6:** Comparison of lightweight 3D MRI models for AD classification (%).

Model	Description	ACC (%)	F1	Params (M)	FLOPs (G)	PD
ResNet3D-Small	Standard small 3D ResNet	91.5 ± 0.4	0.91	1.20	8.73	76.25
MobileNet3D	3D MobileNet variant	92.0 ± 0.3	0.92	0.60	5.10	153.3
ShuffleNet3D	3D ShuffleNet variant	91.8 ± 0.5	0.91	0.55	4.80	167.3
Swin-UNETR	3D Swin Transformer UNET variant	93.7 ± 0.2	0.93	60.12	327.11	1.55
Ours	Proposed lightweight 3D CNN	93.1 ± 0.3	0.93	0.49	4.39	189.87

**Table 7:** Comparison of ADvsNC models (%).

Author	Method	ADvsNC			
		ACC	SEN	SPE	F1 Score
Liu et al. [31]	CNN & 3DDenseNet	88.9	86.6	90.8	-
Bit et al. [32]	SVM	84.1	90.5	-	0.903
Gryshchuk et al. [33]	contrastive self-supervised	82.0	82.0	82.0	-
Hazarika et al. [34]	DenseNet-121	89.3	90.3	-	0.890
ours	3DCNN	91.3	90.0	91.7	0.935

**Table 8:** Comparison of ADvsMCI models (%).

Author	Method	ADvsMCI			
		ACC	SEN	SPE	F1 Score
Zhang et al. [35]	CNN	87.5	86.31	89.23	0.872
Hazarika et al. [34]	DenseNet-121	89.3	89.6	-	0.896
ours	3DCNN	93.4	92.9	93.9	0.932

**Table 9:** Comparison of MCIvsNC models (%).

Author	Method	MCIvsNC			
		ACC	SEN	SPE	F1 Score
Zhang et al. [35]	CNN	85.6	86.4	86.8	0.859
Zhang et al. [36]	GCN	92.7	80.6	89.9	-
Zhang et al. [37]	Transformer & dEC	89.1	91.4	87.2	0.903
Hazarika et al. [34]	DenseNet-121	90.6	90.3	-	0.923
ours	3DCNN	94.4	96.3	93.9	0.972

**Table 10:** Lightweight comparison of models.

Author	Method	Para (M)	FLOPs (G)	MZ (M)	PD
Zhang et al. [35]	CNN	3.42	6.08	-	25.31
Long et al. [38]	CNN	3.35	4.75	-	26.62
Ours	3DCNN	0.49	4.39	1.92	189.87

**Table 11:** Comparative Essay Details.

First author	Datasets	Modality	Preprocessing	Classification
Liu et al. [31]	ADNI	sMRI	ROI extraction	Binary classification
Bit et al. [32]	ADNI	sMRI	ROI extraction	Binary classification
Zhang et al. [35]	ADNI	ADNI	2D slicing	Binary classification
Gryshchuk et al. [33]	ADNI	sMRI	Normalization	Binary classification
Zhang et al. [36]	ADNI	DTI & rs-fMRI	ROI extraction	Binary classification
Zhang et al. [37]	ADNI	sMRI & rs-fMRI	Normalization	Binary classification
Hazarika et al. [34]	ADNI	sMRI	2D slicing	Binary classification
Long et al. [38]	ADNI	sMRI	2D slicing	Binary classification
Ours	ADNI	sMRI	Normalization	Binary classification

## Data Availability

The original data presented in the study are openly available in ADNI at http://adni.loni.usc.edu (accessed on 10 October 2025).
